# Removal of a Retained Percutaneous Nephrostomy Balloon Catheter Using a Trocar Needle: A Case Report

**DOI:** 10.7759/cureus.81437

**Published:** 2025-03-29

**Authors:** Yasuyuki Onishi, Hironori Shimizu, Kaoru Murakami, Takashi Kobayashi, Yuji Nakamoto

**Affiliations:** 1 Diagnostic Imaging and Nuclear Medicine, Kyoto University, Kyoto, JPN; 2 Urology, Kyoto University, Kyoto, JPN

**Keywords:** balloon puncture, catheter removal, complication, non-deflating balloon, percutaneous nephrostomy, trocar needle

## Abstract

While non-deflating Foley catheters are a well-known complication, non-deflating nephrostomy balloon catheters have rarely been reported. We report the case of a 76-year-old man with a non-deflating nephrostomy balloon catheter. To treat this condition, a 19-gauge trocar needle included in an 8.5-F drainage catheter was inserted through the urinary lumen of the catheter to puncture the balloon. The balloon was then deflated, and the nephrostomy catheter was exchanged. Balloon puncture using a trocar needle may be a suitable technique for removing non-deflating nephrostomy balloon catheters.

## Introduction

Percutaneous nephrostomy is an established procedure with a long history [1]. Its indications can be classified into three categories: (1) urinary drainage for urinary tract obstruction; (2) urinary diversion for the treatment of urinary leaks, urinary fistula, and hemorrhagic cystitis; and (3) access to the collecting system for other percutaneous or endoscopic procedures [1-4]. Percutaneous nephrostomy causes several complications: urinary tract infection, sepsis, bleeding, bowel transgression, renal pelvic injury, urinary leakage, catheter dislodgement, and catheter occlusion [4-6]. Complications occur in approximately 10% of patients [1]. Non-deflating nephrostomy balloons are a rare complication of percutaneous nephrostomies and have infrequently been reported. Herein, we report a case of a non-deflating nephrostomy balloon catheter that was successfully removed by puncturing the balloon using a trocar needle advanced through the urinary lumen of the catheter.

## Case presentation

A 76-year-old man had a long-standing history of neuromyelitis optica spectrum disorder with sequelae including muscle weakness in the lower extremities, numbness, and paresthesia below the level of the first lumbar vertebra. One year prior, the patient had been diagnosed with left hydronephrosis. As no tumor or calculus causing hydronephrosis was found, follow-up was performed. Four months prior, he had visited a hospital for hematuria. Cystoscopy revealed no tumor; however, marked trabecular formations and many diverticula were observed. Owing to a high level of residual urine and urinary dysfunction, the patient was diagnosed with neurogenic bladder, for which a Foley catheter was placed in the bladder. One week later, the patient visited the hospital again because urine output from the Foley catheter had stopped. Computed tomography (CT) revealed bilateral hydronephrosis, and his serum creatinine level was 3.27 mg/dL, then the patient was diagnosed with postrenal failure. To relieve postrenal failure, a double-J stent was inserted from the bladder to the right kidney as an initial therapy. Pyelonephritis developed after double-J stent insertion. Furthermore, septic shock secondary to pyelonephritis occurred after replacement of the Foley catheter. The patient was referred to our hospital for the treatment of recurrent pyelonephritis.

**Figure 1:**
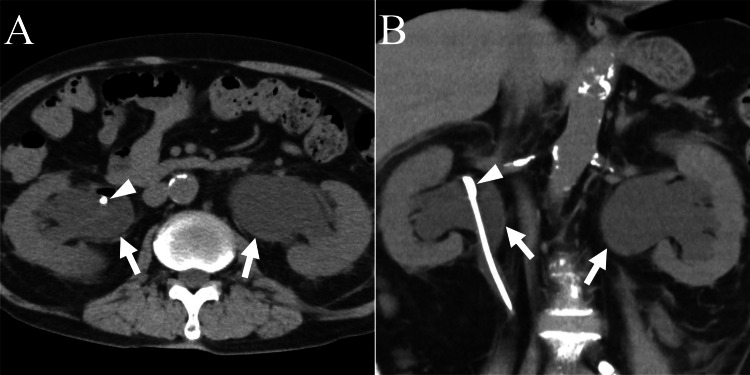
Abdominal computed tomography (CT). Axial (A) and coronal (B) CT images showing bilateral hydronephrosis (arrows). A double-J stent (arrowhead) is observed in the right renal pelvis.

CT at presentation revealed mild bilateral dilatation of the renal pelvis and calyces. His serum creatinine level was 0.89 mg/dL. Cystoscopy showed that the bladder mucosa was edematous with considerable trabecular formations and diverticula. Moreover, the bladder capacity had decreased to <100 mL. We planned an ileal conduit urinary diversion for permanent urine management. Two days later, the patient visited our hospital because of a fever. CT revealed bilateral hydronephrosis (Figures 1A, 1B).

**Figure 2:**
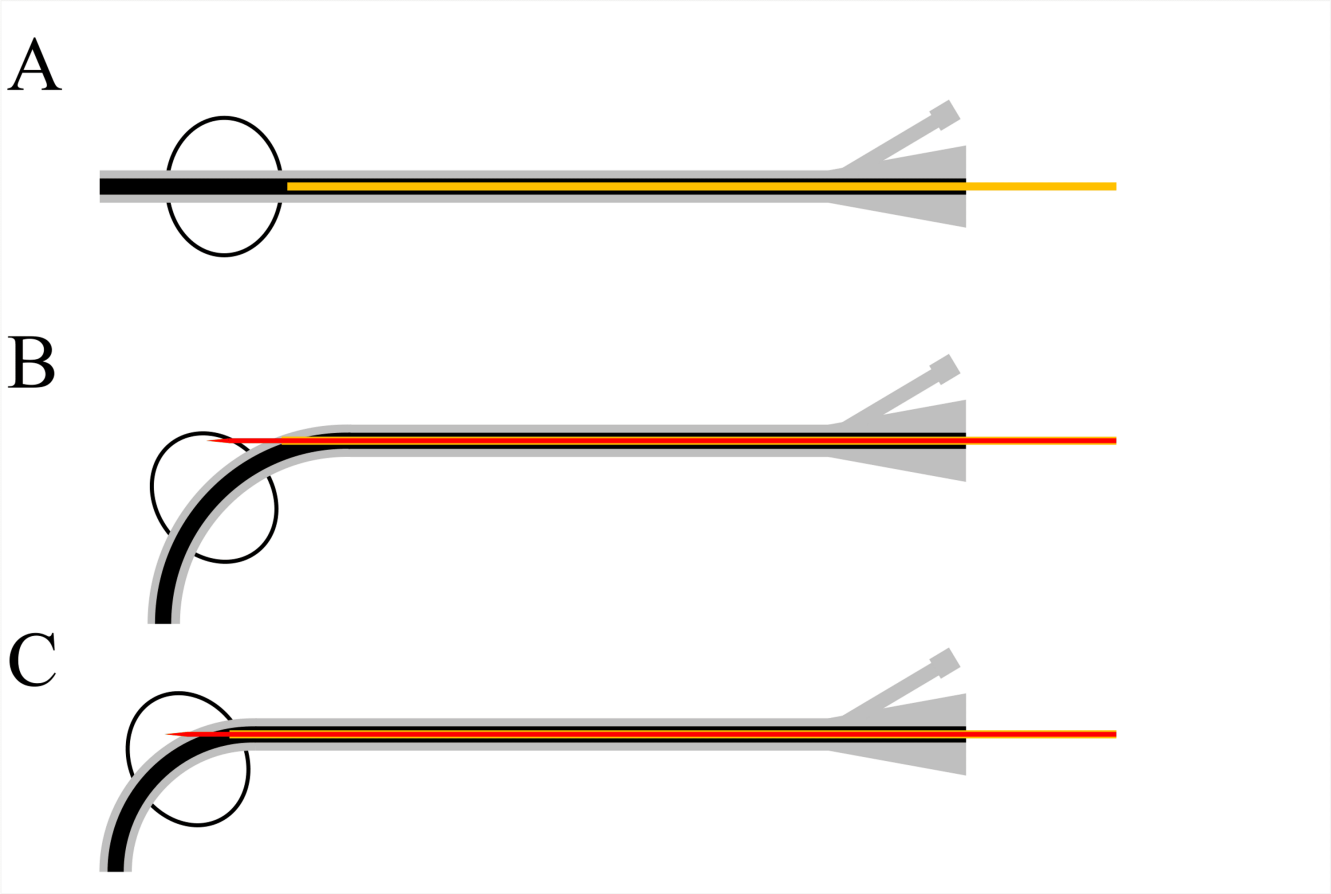
Schematic drawing of experimental balloon puncture procedure using a 19-gauge trocar needle. An 18-gauge metal cannula (yellow) is advanced just before the balloon through the urinary lumen of a nephrostomy catheter (A). The distal part of the nephrostomy catheter is then angulated by gently pushing the distal tip. A 19-gauge trocar needle is then advanced through the cannula, and the urinary lumen wall inside the balloon (B) or balloon wall (C) is punctured. In both cases, the balloon deflated after the puncture. Image Credits: Yasuyuki Onishi

The patient was diagnosed with pyelonephritis and was admitted to our hospital. Emergency bilateral percutaneous nephrostomy was performed using 14-F nephrostomy balloon catheters (Cliny, Tokyo, Japan). A mixture of 1 mL of sterile distilled water and 1 mL of contrast medium was used to inflate the balloons. Septic shock occurred soon after percutaneous nephrostomy placement and intravenous administration of antibiotics was initiated. On the 13th day of hospitalization, the double-J stent was removed after improvement in septic shock. On the 27th day of hospitalization, the urine output from the left nephrostomy catheter stopped. CT revealed that the nephrostomy catheter had dislodged outside the renal pelvis, and the catheter was removed. On the 30th day of hospitalization, ultrasonography showed grade 1 left hydronephrosis and grade 0 right hydronephrosis, according to the Society of Fetal Urology grading system. On the 34th day of hospitalization, an exchange of the right nephrostomy balloon catheter was attempted. However, balloon deflation was unsuccessful. After transection of the balloon port valve, advancement of a 0.035-inch hydrophilic guidewire (Radifocus; Terumo Group, Japan) into the balloon lumen was attempted but was unsuccessful due to crystal deposition in the balloon lumen. The Department of Radiology was consulted for the removal of the retained nephrostomy balloon catheter. We first conducted an experiment outside the body to test this method of deflating the balloon. An 18-gauge metal cannula included in an 8.5-F drainage catheter (Dawson-Mueller Multipurpose Drainage Catheter; Cook Medical, USA) was advanced just before the balloon through the urinary lumen of the nephrostomy catheter. The distal tip of the nephrostomy catheter was manually pushed to angulate the distal portion. A 19-gauge trocar needle included in the drainage catheter was then advanced into the balloon through the nephrostomy catheter. The balloon was punctured by advancing the trocar needle. If the needle hit the urinary lumen wall inside the balloon or the balloon wall, the balloon promptly deflated. A cannula was used to prevent the needle from penetrating the catheter wall. The experimental setup is shown schematically in Figures 2A-2C.

**Figure 3:**
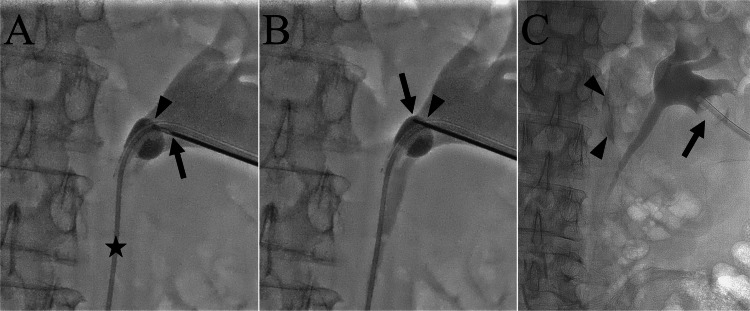
Fluoroscopic images during nephrostomy catheter exchange with the patient in a prone position. (A) An 18-gauge metal cannula (arrowhead) is advanced through the urinary lumen of the nephrostomy catheter close to the balloon. A 19-gauge trocar needle (arrow) is then advanced through the cannula to the balloon. Note that a 4-F catheter (star) is placed in the ureter through the urinary lumen of the nephrostomy catheter and the balloon is inflated with a mixture of contrast medium and saline. (B) The balloon is punctured by advancing the trocar needle (arrow). The arrowhead shows the position of the metal cannula. After this puncture, the balloon deflated. (C) After exchange of the nephrostomy catheter, a new nephrostomy catheter (arrow) is placed. The balloon is inflated using sterile distilled water. Contrast medium (arrowheads) is observed outside the urinary tract, likely due to damage to the renal pelvis caused by the trocar needle.

On the 42nd day of hospitalization, nephrostomy exchange was performed under local anesthesia and moderate sedation. The patient was placed in a prone position, and diluted contrast medium was injected through the urinary lumen of the retained right nephrostomy balloon catheter to dilate the renal pelvis. A 4-F angled catheter and Radifocus were advanced to the proximal ureter, and the Radifocus was removed. The objective of the catheter was to angulate the nephrostomy catheter so that balloon puncture was feasible and to maintain renal pelvis dilation by injecting contrast medium. An 18-gauge metal cannula was then advanced through the urinary lumen of the nephrostomy catheter close to the balloon (Figure 3A). Next, a 19-gauge trocar needle was advanced through the cannula, and the balloon was punctured using the needle (Figure 3B). After the third puncture, the balloon deflated, and the nephrostomy catheter was exchanged to a new 14-F nephrostomy balloon catheter using a 0.035-inch steel guidewire (Fixed Core Wire Guide; Cook Medical) (Figure 3C). Sterile distilled water (2 mL) was used to inflate the balloon. During balloon puncture, the contrast medium spread outside the renal pelvis because of renal pelvis injury by the trocar needle. As no signs of vascular injury such as hematuria were observed, treatment was considered unnecessary. After the procedure, the patient developed a fever of around 38°C, which responded well to the intravenous administration of antibiotics. During hospitalization, the patient’s daily life activities declined. After rehabilitation, the patient was discharged on the 56th day of hospitalization.

## Discussion

Balloon deflation failure is a well-known complication of Foley catheters [7-9]. The use of fluids other than sterile distilled water for balloon inflation can lead to solute crystallization, causing occlusion of the balloon lumen [7]. In the present case, the use of saline and contrast medium admixture was considered the cause of the non-deflating balloon. Distilled water must be used when dilating the balloon.

Several methods to remove a non-deflating Foley catheter have been reported, including removal of the valve mechanism by cutting the balloon port, advancing the sharp end of a ureteral stent stylet through the balloon lumen, and puncturing the balloon through the suprapubic, transvaginal, transurethral, and transperineal route [8,10]. In contrast, reports on non-deflating nephrostomy balloon catheters are sparse. Anderson and Monga reported the successful removal of a non-deflating nephrostomy balloon catheter by puncturing the balloon using an 18-gauge Chiba needle advanced through the puncture site directly over the nephrostomy catheter under fluoroscopic guidance [11]. However, this method carries the risk of needle penetration of the tract around the catheter, damaging the renal parenchyma. Vascular or peel-away sheaths can be used to remove an obstructed pigtail nephrostomy catheter [12,13]. In this technique, a vascular or peel-away sheath of the same size or larger is advanced over a retained nephrostomy catheter, which is then pulled out. This technique was considered for the removal of the non-deflating nephrostomy balloon catheter; however, it was not selected because it includes dilatation of the catheter tract using a sheath, which may damage the renal parenchyma around the tract. Another option for nephrostomy catheter removal in the present case was to puncture the balloon with a needle through the renal parenchyma under fluoroscopy or ultrasound guidance, as has been reported for Foley catheters. However, we preferred to puncture the balloon without passing through the renal parenchyma and inserted a trocar needle through the balloon lumen. This method seemed safer and less invasive than those previously reported and can also be used to remove balloon catheters placed at other sites. However, this method involves the use of a long metal needle, which may be difficult if the catheter pathway is not straight.

In the present case, needle injury to the renal pelvis occurred as a complication. We believe that this was caused by the needle penetrating the balloon and contacting the renal pelvis behind it. During balloon puncture with the needle, we should have rotated the x-ray tube so that the needle and balloon could be seen in an end-on view, which may have prevented this complication.

## Conclusions

We report a case of a non-deflating nephrostomy balloon catheter. The use of saline and contrast medium admixture for balloon inflation caused balloon lumen occlusion. Distilled water should be used for balloon inflation. The non-deflating balloon catheter was successfully removed by puncturing the balloon using a trocar needle advanced through the urinary lumen. This technique has the advantage of not traversing the renal parenchyma and may be suitable for removing non-deflating nephrostomy catheters.
